# Data on items of AKUSSI in Alkaptonuria collected over three years from the United Kingdom National Alkaptonuria Centre and the impact of nitisinone

**DOI:** 10.1016/j.dib.2018.09.021

**Published:** 2018-09-12

**Authors:** R. Griffin, E.E. Psarelli, T.F. Cox, M. Khedr, A.M. Milan, A.S. Davison, A.T. Hughes, J.L. Usher, S. Taylor, N. Loftus, A. Daroszewska, E. West, A. Jones, M. Briggs, M. Fisher, M. McCormick, S. Judd, S. Vinjamuri, N. Sireau, J.P. Dillon, J.M. Devine, G. Hughes, J. Harrold, G.J. Barton, J.C. Jarvis, J.A. Gallagher, L.R. Ranganath

**Affiliations:** a Liverpool Cancer Trials Unit, University of Liverpool, Block C, Waterhouse Building, Liverpool L69 3GL, UK; b Department of Clinical Biochemistry and Metabolic Medicine, Royal Liverpool University Hospital, Prescot Street, Liverpool L7 8XP, UK; c Department of Physiotherapy, Royal Liverpool University Hospital, Prescot Street, Liverpool L7 8XP, UK; d Department of Rheumatology, Royal Liverpool University Hospital, Prescot Street, Liverpool L7 8XP, UK; e Department of Dermatology, Royal Liverpool University Hospital, Prescot Street, Liverpool L7 8XP, UK; f Department of Anaesthesia, Royal Liverpool University Hospital, Prescot Street, Liverpool L7 8XP, UK; g Department of Ophthalmology, Royal Liverpool University Hospital, Prescot Street, Liverpool L7 8XP, UK; h Department of Cardiology, Royal Liverpool University Hospital, Prescot Street, Liverpool L7 8XP, UK; i Department of ENT, Royal Liverpool University Hospital, Prescot Street, Liverpool L7 8XP, UK; j Department of Dietetics, Royal Liverpool University Hospital, Prescot Street, Liverpool L7 8XP, UK; k Department of Nuclear Medicine, Royal Liverpool University Hospital, Prescot Street, Liverpool L7 8XP, UK; l AKU Society, 66 Devonshire Road, Cambridge, UK; m Department of Musculoskeletal Biology, University of Liverpool, L69 7ZX, UK; n Department of Psychological Sciences, University of Liverpool, L69 7ZX, UK; o School of Sport and Exercise Science, Liverpool John Moores University, Liverpool, UK

## Abstract

Alkaptonuria is a rare genetic disorder characterized by a high level of circulating (and urine) homogentisic acid (HGA), which contributes to ochronosis when it is deposited in connective tissue as a pigmented polymer. In an observational study carried out by National AKU Centre (NAC) in Liverpool, a total of thirty-nine AKU patients attended yearly visits in varying numbers. At each visit a mixture of clinical, joint and spinal assessments were carried out and the results calculated to yield an AKUSSI (Alkaptonuria Severity Score Index), see “Nitisinone arrests ochronosis and decreases rate of progression of Alkaptonuria: evaluation of the effect of nitisinone in the United Kingdom National Alkaptonuria Centre” (Ranganath at el., 2018). The aim of this data article is to produce visual representation of the change in the components of AKUSSI over 3 years, through radar charts. The metabolic effect of nitisinone is shown through box plots.

**Specifications table**

**Table:** 

Subject area	Rare inborn error of metabolism
More specific subject area	Tyrosine pathway disorder, Alkaptonuria, AKUSSI, severity, nitisinone, homogentisic acid, natural history
Type of data	Radar charts and box plots
How data was acquired	Clinical assessments including subjective pain scoring, photographs, history, ultrasound abdomen, echocardiogram, dual energy x-ray absorptiometry, x-ray spine and PET-CT scan
Data format	Raw data
Experimental factors	Observations were made over 3 years before nitisinone and over 3 years after in a cohort of patients with Alkaptonuria.
Experimental features	Assessments and investigations were carried out over 5 visits to derive numerical scores, which were then used to calculate the Alkaptonuria Severity Score Index (AKUSSI).
Data source location	National AKU Centre (NAC) in Liverpool, UK
Data accessibility	Data is available in this manuscript
Related research article	Ranganath LR, Khedr M, Milan AM, Davison AS, Hughes AT, Usher JL, Taylor S, Loftus N, Daroszewska A, West E, Jones A, Briggs M, Fisher M, McCormick M, Judd S, Vinjamuri S, Griffin R, Psarelli EE, Cox TF, Sireau N, Dillon JP, Devine JM, Hughes G, Harrold J, Barton GJ, Jarvis JC, Gallagher JA. Nitisinone arrests ochronosis and decreases rate of progression of Alkaptonuria:evaluation of the effect of nitisinone in the United Kingdom National Alkaptonuria Centre. Molecular Genetics and Metabolism 2018 (in press) [1].

**Value of the data**

• *Actual data is shared on the components of the AKUSSI* **–** This data will show a visualisation of how the AKUSSI is composed.
• *This is the first time such data is being made available in relation to nitisinone therapy* **–** Researchers/Scientific community will be able to see the impact nitisinone therapy is having on AKUSSI for patients with Alkaptonuria.
• *Data is shown both before and after nitisinone administration* **–** This data is provided to show the comparison of the AKUSSI before and after nitisinone therapy.

## 1 Data

The Alkaptonuria Severity Score index (AKUSSI) was developed to allow quantification of disease morbidity and thus allow it to be used to track changes in disease as well as potential therapies. A large number of items were characterized by clinical assessment to derive the Clinical, Joint and Spine AKUSSI, the sum of which is the ALL AKUSSI. The data contains each component of the AKUSSI over 5 visits, both before and after nitisinone therapy.

Fig. 2, Fig. 3, Fig. 4 show the metabolic effect nitisinone has on patients with Alkaptonuria, through boxplots. While Fig. 5, Fig. 6, Fig. 7, Fig. 8, Fig. 9, Fig. 10, Fig. 11, Fig. 12 show the components of AKUSSI broken down into radar charts and the scores at each medical visit with the intervention of nitisinone.

**Fig. 1:**
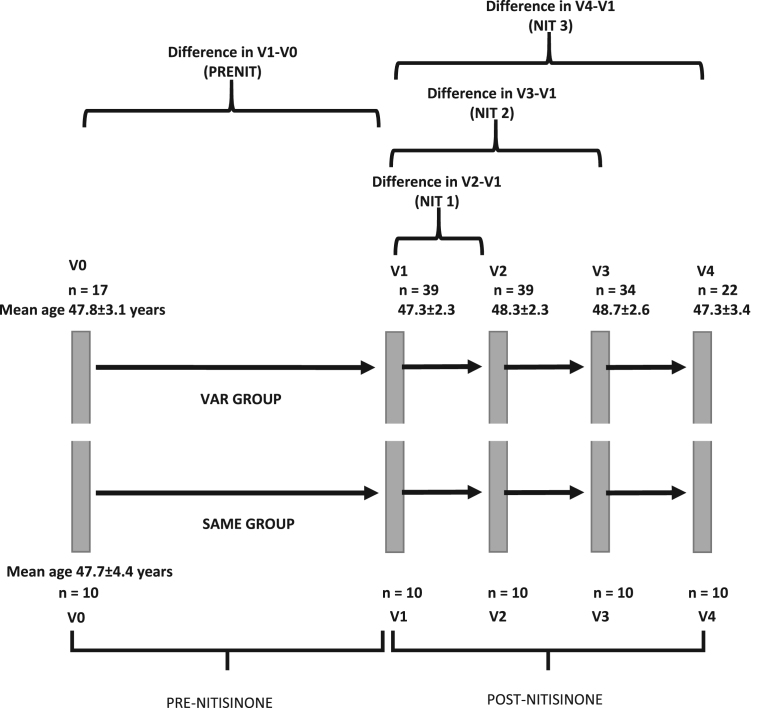
Plan of the National Alkaptonuria Service: *The VAR group V0 visit consisted of the 10 patients from the SAME group plus seven additional patients who attended the NAC twice without receiving nitisinone. The SAME refers to ten patients attending the research study between 2008 and 2011. The V1, V2, V3 and V4 refer to yearly visits to the NAC. The NIT 1, NIT 2 and NIT 3 refer to change scores per patient per year after one, two and three years of nitisinone therapy. The numbers of patients in each group, their mean age and years of follow-up are also shown in the figure.

**Fig. 2:**
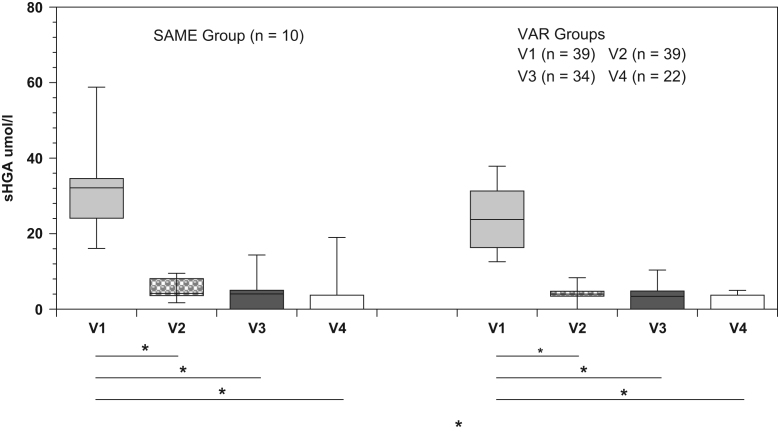
s-HGA concentrations in SAME and VAR groups, Pre- and Post-Nitisinone. Scores are shown as box plots with and interquartile range. The level of significance of results is shown as *p < 0.001.

**Fig. 3:**
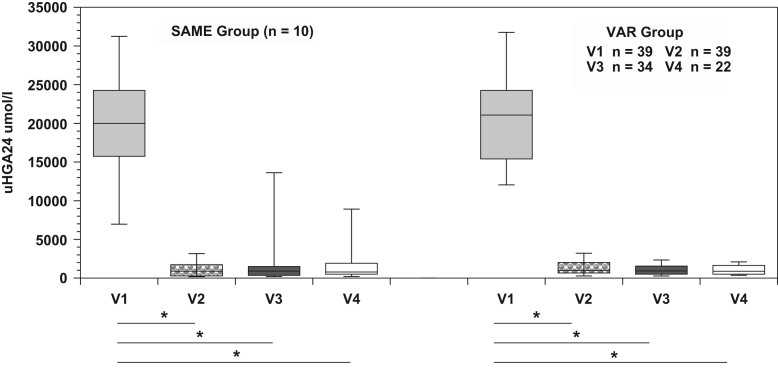
u-HGA_24_ concentrations in SAME and VAR groups, Pre- and Post-Nitisinone. Scores are shown as boxplots with interquartile range. The level of significance of results is shown as *p < 0.001.

**Fig. 4:**
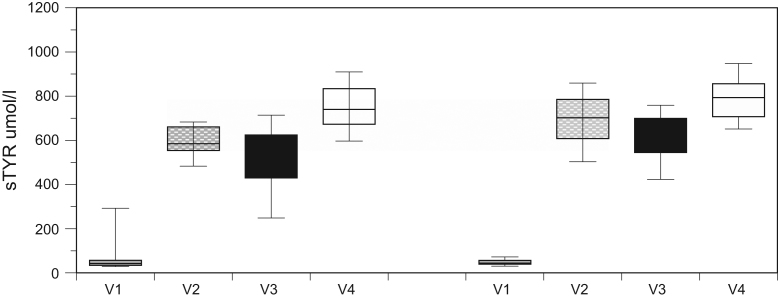
s-TYR concentrations in SAME and VAR groups, Pre- and Post-Nitisinone. Scores are shown as boxplots with interquartile range. The level of significance of results is shown as *p < 0.001.

**Fig. 5:**
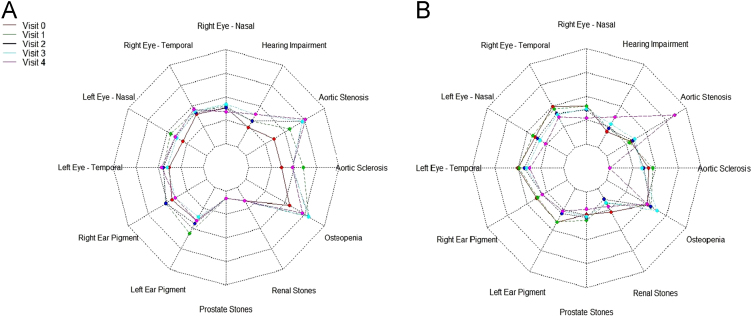
Radar chart showing 12 of the overall clinical AKUSSI components. The graph on the left (A) is data from the SAME group dataset and shows the mean score per variable over 5 visits (including baseline). The right graph (B) shows the same information but for the VAR group.

**Fig. 6:**
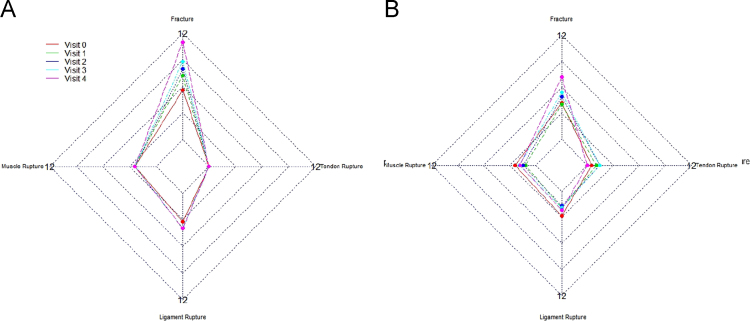
Radar chart showing 4 of the overall clinical AKUSSI components. The graph on the left (A) is data from the SAME group dataset and shows the mean score per variable over 5 visits (including baseline). The right chart (B) shows the same information but for the VAR group.

**Fig. 7:**
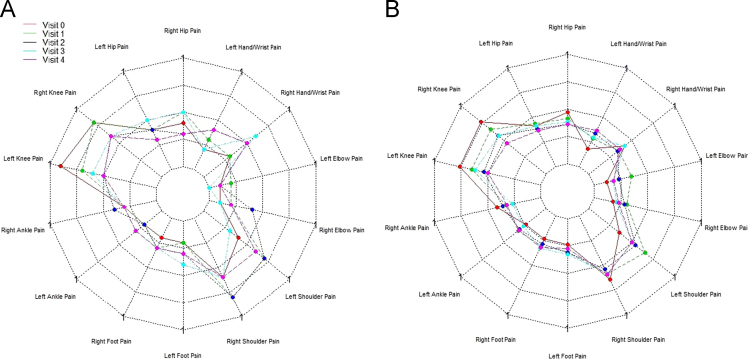
Radar chart showing the 14 large joint areas that make up the joint pain AKUSSI score. The graph on the left (A) is data from the SAME group dataset and shows the mean score per variable over 5 visits (including baseline). The right chart (B) shows the same information but for the VAR group.

**Fig. 8:**
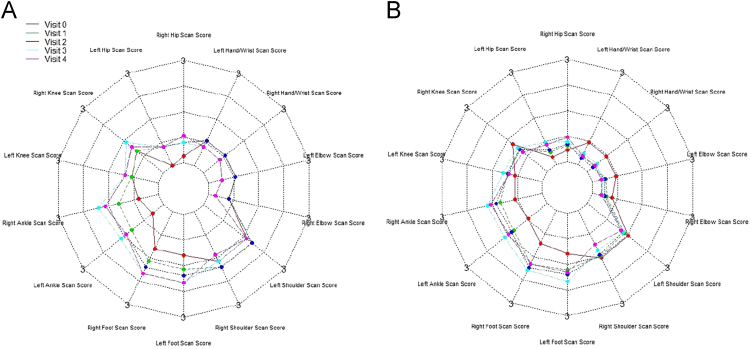
Radar chart showing the 14 large joint areas that make up the Scintigraphic scan joint AKUSSI score. The graph on the left (A) is data from the SAME group dataset and shows the mean score per variable over 5 visits (including baseline). The right chart (B) shows the same information but for the VAR group.

**Fig. 9:**
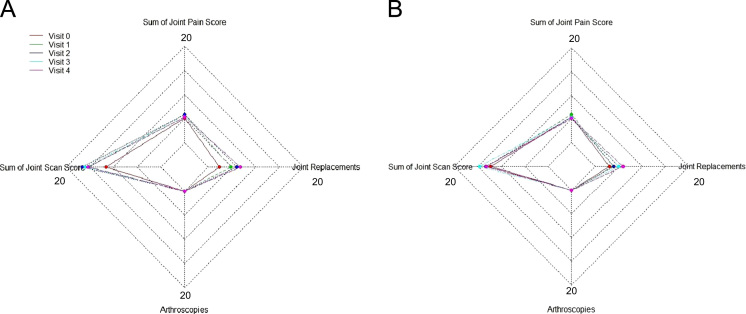
Radar chart showing the 4 components of the overall joint AKUSSI score. The graph on the left (A) is data from the SAME group dataset and shows the mean score per variable over 5 visits (including baseline). The right chart (B) shows the same information but for the VAR group.

**Fig. 10:**
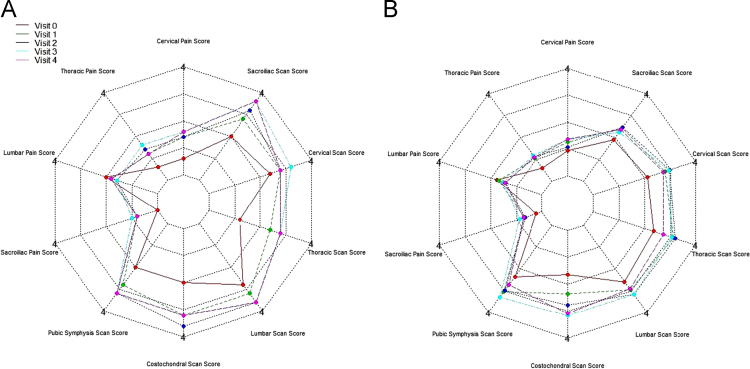
Radar chart showing the 4 components of the spinal AKUSSI pain score and the 6 components of the Scintigraphic scan AKUSSI pain score. The graph on the left (A) is data from the SAME group dataset and shows the mean score per variable over 5 visits (including baseline). The right chart (B) shows the same information but for the VAR group.

**Fig. 11:**
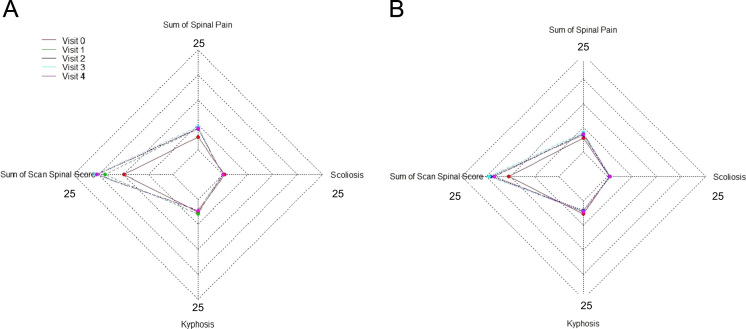
Radar chart showing the 4 components of the overall spine AKUSSI score. The graph on the left (A) is data from the SAME group dataset and shows the mean score per variable over 5 visits (including baseline). The right chart (B) shows the same information but for the VAR group.

**Fig. 12:**
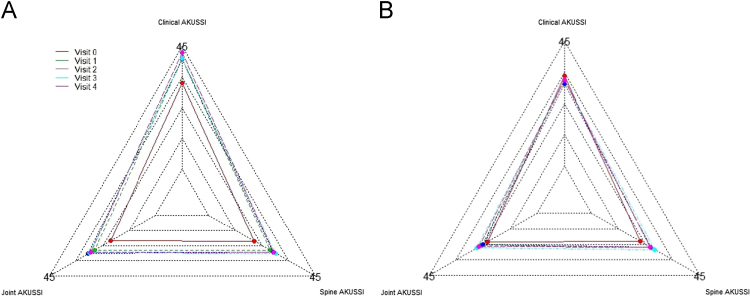
Radar chart showing the 3 overall components of the AKUSSI score. The graph on the left (A) is data from the SAME group dataset and shows the mean score per variable over 5 visits (including baseline). The right chart (B) shows the same information but for the VAR group.

## 2 Experimental design, materials and methods

Overall, thirty-nine AKU patients attended the National AKU Centre (NAC) in Liverpool (Fig. 1). Varying numbers attended yearly visits leading to creation of two datasets The VAR group (variable numbers of patients at each visit) and the SAME group (the same ten patients who attended all of the visits) (described in more detail in accompanying main manuscript). Nitisinone 2 mg was commenced at baseline (V1) and systematic assessments were carried out at all visits.

The VAR group at visit V0 had the 10 patients from the SAME group plus 7 additional patients, who visited the NAC before dosing. From here forward, the SAME group consists of the same 10 patients and the VAR group consists of the SAME group as well as an additional 29, 29, 24 and 12 patients at visits V1, V2, V3 and V4 respectively.

Assessments (AKUSSI) were carried out at V0 (pre-baseline), V1, V2, V3 and V4. Eye and ear ochronosis, calculi (renal and prostate), osteopenia, fracture, ruptures (muscle, ligament and tendon), aortic valve disease and hearing impairment collectively are components of the CLINICAL AKUSSI category. Pain and scintigraphic scan joint score of 14 large joint areas (includes hip, knee, ankle, shoulder, hand/wrist, foot and elbow), arthroscopy and joint replacements, comprise the JOINT category. Spinal pain score (cervical, thoracic and sacroiliac), Scintigraphic scan spine score (pubic symphysis, costochondral, lumbar, thoracic, cervical and sacroiliac), kyphosis and scoliosis comprise the SPINE category. The overall AKUSSI score is calculated over the CLINICAL, JOINT and SPINE scores.

In order to produce a visualisation of the AKUSSI assessment results and illustrate the effect nitisinone has over time, radar charts were produced. A radar chart is a graphical method used for displaying multivariate data, represented on axes starting from the same point. Each spoke on the chart represents one variable and the 5 different plots on each spoke, represent the mean of the data at each of the 5 visits (V0, V1, V2, V3, and V4). HGA was measured on acidified 24 h urine (u-HGA_24_) and acidified serum (s-HGA) samples from each visit as previously described by tandem mass spectrometry.

The metabolic effect has been captured visually through bar charts and statistically analyzed by Student׳s ‘t’ test (paired for the SAME group and unpaired for the VAR group).

## 3 Nitisinone effect on metabolic measurements

The effect of nitisinone on serum and 24 h urine HGA both in SAME and VAR groups, as well as serum tyrosine in SAME and VAR groups is shown in Fig. 2, Fig. 3, Fig. 4.

## 4 Visualisation of AKUSSI components over time

The radar charts displayed below show different components of the AKUSSI assessment. In each figure the same components are shown for the SAME group (left chart, A) and also for the VAR group (right chart, B). As the VAR group consists of a mixture of participants, it is included for validation purposes. Each radar chart displays the upper range score around the outside of the graph, while each web line represents a percentage of the total score.

An improvement in mean score between the later visit times for the scintigraphic ankle and knee scan score is seen in Fig. 8 (left chart). This can also be seen in Fig. 8 (right chart).
