# Supplementary material for: Data on items of AKUSSI in Alkaptonuria collected over three years from the United Kingdom National Alkaptonuria Centre and the impact of nitisinone
Source: Data Brief. 2018 Sep 12;20:1620–8. doi: 10.1016/j.dib.2018.09.021 (PMC6157456; doi:10.1016/j.dib.2018.09.021)
Supplement: Supplementary file 1 — Supplementary material [file mmc1.docx]

2^nd^ September 2018

The Editor

Molecular Genetics and Metabolism

Dear Sir/madam

I submit the revised Data in Brief manuscript on behalf of my colleagues (and my co-authors) for consideration of publication in the Molecular Genetics and Metabolism journal.

The article has been revised as suggested.

There are no conflicts to declare. The data has not been published elsewhere.

Yours sincerely


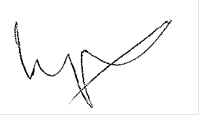


Professor Lakshminarayan Ranganath

Clinical Director of the National Alkaptonuria Centre, UK

**Instructions when you submit your Data in Brief with your research article**

1. Complete this template below. Make sure you reference all data files directly in this template at the appropriate point.

***We have refenced the data files directly in this template as required.***

1. Zip this completed Word document and any data files relevant to the Data in Brief (whichever supplementary data files you have chosen to include) into a single. Zip file. When you submit your revised research article, please upload this .zip file as a “Data in Brief” item.

***We have this single file as the data in brief submission.***

1. Double check in your research article that any reference to supplementary files that have been converted to your Data in Brief article now properly reference the Data in Brief instead. i.e. (see supplementary Figure 1) should now say, (see Figure 1 in Ref [#]) where your Data in Brief article is included in the reference list of your research article (including title, authors, journal name *Data in Brief* and the text “*submitted”*)

***The refencing of the figures do not use the term supplementary. The research article is referenced .***

1. Make sure you reference your associated research article in the reference list here as well. You may reference this as “in press”

***We have indicated this.***

1. ***We have removed the interpretation of the figures as requested. Only the legend stating what the data refers to is shown.***
